# Training needs of professional nurses in primary health care in the Cape Metropole, South Africa

**DOI:** 10.4102/phcfm.v15i1.3741

**Published:** 2023-05-16

**Authors:** Ashley Kordom, Felicity Daniels, Jennifer Chipps

**Affiliations:** 1 School of Nursing, Faculty of Community and Health Sciences, University of the Western Cape, Cape Town, South Africa

**Keywords:** training needs, professional nurses, primary health care, nursing, education

## Abstract

**Background:**

In the fast-changing healthcare environment, it is important to ensure that primary health care (PHC) nurses are suitably qualified and have access to appropriate and relevant ongoing education.

**Aim:**

The aim of this study was to determine the training needs of professional nurses working in PHC facilities.

**Setting:**

The research was conducted in PHC facilities in the Cape Metropole, Western Cape, South Africa.

**Methods:**

A quantitative descriptive survey with all-inclusive sampling was used. All professional nurses (*N* = 303), employed for at least a minimum of 6 months in PHC facilities were included in the study. The Hennessy–Hicks Training Needs Analysis (TNA) questionnaire was used to collect the data on professional tasks training needs and open-ended questions for specific PHC contextual training needs. Importance and performance means for each of the TNA subsections and training needs were calculated. Open-ended questions were analysed using content analysis, identifying training domains and topics in terms of frequency and ranking.

**Results:**

The TNA identified ‘Research’ as the highest training need. Research tasks were significantly rated as the least important and the lowest rated performance compared to other domains. Child mental health was rated as the most important specific training need.

**Conclusion:**

The results of this survey provide insight into the training needs of professional nurses employed in PHC facilities and highlight the need for child mental healthcare and research training in this setting.

**Contribution:**

The study contributes to the understanding of the training and education needs of professional nurses working in PHC facilities.

## Introduction

South Africa is experiencing a quadruple burden of disease with high rates of human immunodeficiency virus (HIV), acquired immunodeficiency syndrome (AIDS) and tuberculosis (TB); maternal and child mortality; hypertension and cardiovascular diseases, diabetes, cancer, mental illnesses and chronic lung diseases; and injury and trauma.^1^ All these conditions are initially managed at primary health care (PHC) level, as this is the first level of contact for individuals within the three-tiered health system, bringing healthcare as close as possible to where people live and work.^2^ Primary health care is globally acknowledged as the foundation of universal health coverage^3^ and South Africa is committed to the renewed Alma Ata Declaration and the Astana Declaration for PHC with a focus on equity, equality and all-inclusive health coverage.^4^

In South Africa, PHC is predominantly nurse-led and doctor-supported and offered in community health clinics, day and community health centres (CHCs).^5^ Nurses in these settings are the frontline healthcare providers rendering about 90% of preventative and curative services including chronic disease management.^6^ The services at PHC facilities include inter alia maternal and child care, immunisations, family planning and care for chronic illnesses, usually managed by professional nurses employed in PHC facilities. These nurses include professional nurses who have successfully completed a nursing diploma or degree that allows for registration under South African Nursing Council Regulation R425 as nurse (General, Psychiatric and Community) and Midwife, in this study referred to as non-specialist in PHC, as well as professional nurses who have successfully completed a post-basic specialisation in Clinical Nursing Science, Health Assessment, Treatment and Care register under Regulation R48 of the South African Nursing Council, referred to as specialist in PHC. With the recent integration of mental health into PHC, this is often the first point of contact for patients with mental health problems, including children.^7^ During 2016, in the Western Cape, 8300 (4.4%) of the 188 369 patients, who were seen in PHC facilities, were children with mental health problems.^8^ In today’s fast-changing healthcare environment,^9^ the complexity of the disease profiles of the patients attending PHC facilities, and the fact that the PHC centre is the first contact for most patients, requires a current, competent and specialised nursing workforce. It is, therefore, evident that nurses have a central role to play in providing quality patient care to patients presenting at PHC facilities, while ensuring that they keep abreast of the latest developments.^10^

Currently, in South Africa, in response to the need, a post-basic specialisation in Clinical Nursing Science, Health Assessment, Treatment and Care is offered by nursing education institutions and national strategies such as the National Core Standards^11^ and the Ideal Clinic have been developed and implemented^12^ to improve delivery of PHC services. In addition, quality improvement strategies such as integrated management of childhood illness (IMCI),^13^ practical approach to care kit (PACK)^14^ and basic antenatal care (BANC)^15^ have all been introduced in PHC. Despite these quality improvement strategies, PHC nurses still report challenges with legislation, policy and guidelines,^12,16^ inadequate support and mentoring,^16,17^ non-standardised training and inadequate training and awareness.^13,16,17^

Reported challenges highlight the need for a training strategy to ensure provisioning for the training of a specialised workforce in PHC and Continuing Professional Development (CPD) opportunities for professional nurses working in PHC facilities. Such a strategy should be based on a training needs assessment and should address the professional nursing tasks and the context-specific training needs of professional nurses in this setting.^18^ In global studies of the training needs of nurses, where the standard Training Needs Analysis (TNA) of professional tasks was used, it has been recommended that a TNA should be conducted as the first step in designing a CPD strategy.^19^ The South African Nursing Council has recently finalised their CPD framework for all nurses and midwives but it has not yet been implemented.^20^ The absence of a formal TNA can result in unnecessary expenditure on inappropriate and outdated CPD programmes^19^ and curricula for PHC nurses that do not respond to the healthcare needs of society.

The TNA tool, particularly for nurses in both developed and developing countries, has been underutilised.^10^ The results of research studies^18,21,22,23,24^ done on TNA of nurses, consistently showed that research was identified as one of the highest training needs subcategories. Similarly, a seminal work by Hennessy and Hicks showed that United Kingdom health visitors and district nurses and Australian hospital nurses had the highest training needs in terms of collecting and collating relevant research information.^25^ A research study conducted in England among healthcare professionals including doctors, nurses and managers reported on the least important and lowest performance research-related tasks being identifying viable research topics, designing research studies, accessing resources to undertake research, that is, money, information, equipment, securing time to undertake research.^26^ Furthermore, other training needs, such as patient care, communication and management, are reported in other studies as subcategories that also require attention.^10,27^

Most published TNA studies have been conducted in high-income countries.^19,21,22,24,26^ Though some studies of TNA have been published in low- and middle-income countries,^10,23,27^ including South Africa,^28^ continuous education of nurses working in PHC has not received necessary attention and support.^10^ Specifically, there is a paucity of literature on the training needs of professional nurses working in PHC facilities in South Africa. Therefore, the aim of this study was to determine the training needs of professional nurses working in PHC facilities in the Cape Metropole, Western Cape, South Africa. To address this gap and to inform the development of relevant, evidence-based, context-specific training for PHC nurses in South Africa, a survey of training needs was conducted with nurses in PHC facilities in the Cape Metropole, Western Cape, South Africa.

## Research methods and design

### Study design

A quantitative descriptive survey was conducted to assess the training needs of professional nurses employed at PHC facilities in two of the four health substructures in the Cape Metropole, Western Cape, South Africa. The two health substructures were purposefully selected because they have the largest population of professional nurses (approximately 400) that provide healthcare to a diverse group of patients at these PHC facilities.

### Setting

The Western Cape, one of the nine provinces in South Africa, was estimated to have a population of 7 million people in 2020/2021 and approximately 80% of the population made use of the public health services.^29^ The Western Cape is divided into six health subdistricts, namely Central Karoo, Eden, Cape Winelands, Cape Metropole, Overberg and West Coast.^29^ The Cape Metropole incorporates four health substructures, namely Southern Western substructure, Klipfontein Mitchell’s Plain substructure, Khayelitsha Eastern substructure and Northern Tygerberg substructure.^29^ Healthcare facilities, such as primary, district and tertiary healthcare facilities, are found within these areas.^29^ At the time of the study, PHC facilities comprised 10 CHCs, 45 community day centres (CDCs) within the provincial government and 72 clinics within the local government Department of Health (DoH).^29^ At PHC facilities, almost 10m contacts occurred during the 2020/2021 financial year.^29^

### Study population and sampling strategy

The study population comprised an accessible population of 303 professional nurses working in PHC facilities in the Cape Metropole, Western Cape, South Africa. All-inclusive sampling was used, and all professional nurses employed in PHC facilities for more than six months were invited to participate in the study.

### Data collection

Gate keeper permission was obtained from the PHC clinic manager. The researcher met with the PHC facility managers in November 2019 to discuss the data collection process. Data collection commenced in January 2020, but due to the coronavirus disease 2019 (COVID-19) interruptions, it was only completed in August 2021. Data were collected face-to-face in English, which is the medium of instruction in healthcare facilities in South Africa. All ethical principles were maintained through data collection. On the day of data collection, the researcher provided eligible respondents with an information sheet and verbally explained the study. The respondents signed written consent which was separated from the questionnaire to allow for anonymity. Further to ensure anonymity, each respondent was provided with an envelope to insert the completed questionnaires, which the researcher collected a week later.

### Data collection instrument

The Hennessy–Hicks TNA questionnaire,^30^ one of the most widely used validated tools, was used to collect the data.^31^ The Hennessy–Hicks TNA questionnaire was deemed appropriate for this study because it identifies the training needs and outcomes of targeted continuous professional development.^31^ Furthermore, the Hennessy–Hicks TNA questionnaire is widely used as a clinical practice and educational quality improvement tool across continents.^31^ Permission was obtained from the author to use the questionnaire. The self‑administered questionnaire consists of 5 subsections with 30 items. The subsections comprise: nine items for research and audit (items 3, 6, 7, 9, 15, 21, 25, 26, 28); six items for communication and teamwork (items 1, 5, 8, 13, 14, 27); six items for clinical task (items 10, 12, 17, 18, 22, 24); three items for administration (items 2, 20, 29) and six items for management and supervisory tasks (items 4, 11, 16, 19, 23, 30) asking questions on importance and performance. Each item on the questionnaire is rated along a seven-point scale in two different ways, namely how important a task is to the respondent’s job (rating A) and how well the task is performed by the respondent at the time of rating (rating B). A pre-test of the Hennessy–Hicks TNA questionnaire was conducted with 12 professional nurses employed in PHC facilities in one of the health substructures where the study was conducted. There were no changes to the Hennessy–Hicks TNA questionnaire and the responses from the professional nurses were, therefore, also included in the main study. The questionnaire has well-established reliability with strong internal consistency, which was also shown in this study (Table 1).

**TABLE 1:** Reliability of subscales.

Subscales	Number of items	Items	Importance	Performance	Training need
Research/audit	9	3, 6, 7, 9, 15, 21, 25, 26, 28	0.095	0.947	0.953
Communication/teamwork	6	1, 5, 8, 13, 14, 27	0.865	0.869	0.836
Clinical tasks	6	10, 12, 17, 18, 22, 24	0.877	0.833	0.823
Administration	3	2, 20, 29	0.734	0.699	0.726
Management/supervisory task	6	4, 11, 16, 19, 23, 30	0.845	0.861	0.839
Overall Cronbach’s alpha (α)	30	1–30	0.096	0.952	0.959

In addition, to identify the specific PHC training needs, the respondents were asked to describe five specific training needs related to their current practice and to rank these needs from ‘4’ (most important) to ‘1’ (least important).

### Data analysis

The data were captured, cleaned and analysed using the International Business Manual (IBM) Statistical Package for Social Science (SPSS) version 27. Two categories of training needs were assessed: training needs based on the standard TNA of professional nursing tasks using a quantitative descriptive survey and specific training needs for PHC using open-ended written questions. Descriptive statistics were calculated for the demographics and means and 95% confidence intervals (CIs) for the performance and importance of training items. Training needs were calculated for each item by subtracting the performance from the importance mean score. Differences between specialist in PHC and non-specialist in PHC were measured using chi-square test (χ^2^) and Mann–Whitney *U* test. Significance was set at *p* < 0.05. The open-ended questions were analysed using content analysis with identification and frequency calculation of domains of training needs and topics. Responses were coded and similar codes were grouped into domains using current services provided in PHC facilities as the structure to generate broad training needs from the categories. The co-researchers reviewed the coding, confirmed the domains to ensure that it represented the analysed data. Once consensus was reached, two types of analysis were done by the researchers. An overall score per domain based on ranking score of most important was calculated and the frequency of rating for the overall topics listed was calculated.

### Ethical considerations

Ethical clearance was obtained from the Biomedical Research Ethics Committee of the University of the Western Cape, the Provincial Government Western Cape and Department of Health and City Council Research Ethics Committee (local government). Principles highlighted in the Declaration of Helsinki were adhered to.^32^ Biomedical Research Ethics Committee of the University of the Western Cape BMREC registration number: 19/8/10, Provincial Government Western Cape, Department of Health Reference: WC_201910_021 City Council Research Ethics Committee Reference number: 8231.

## Results

### Demographics

A total of 205 professional nurses employed in PHC facilities out of a total of 303 completed the questionnaires (67.7% response rate). The average age of the respondents was 38.5 years (± 9.6 years) with the age ranging from 23 to 63 years. Most of the respondents were females (188, 91.7%). More than half (*n* = 119, 58.0%) of the respondents reported their highest qualification as either a basic nursing diploma (*n* = 66, 32.2%) or degree (*n* = 53, 25.8%) that allowed them to register as a Nurse (General, Psychiatric and Community) and Midwife as per Regulation R425 of the South African Nursing Council. Eighty-three (40.5%) of the respondents reported that they had a post-basic qualification in nursing and only three (1.5%) had a master’s degree in nursing. Although 86 (42.0%) respondents identified themselves as clinical nurse specialists, only 72 (35.1%) respondents were registered with the South African Nursing Council under Regulation R48 for Clinical Nursing Science, Health Assessment, Treatment and Care, the PHC specialist post-basic qualification. The remaining 13 clinical nurse specialist who were registered with the South African Nursing Council according to Regulation Number R212 included post-basic psychiatric nursing science (2%), occupational health nursing (1.5%), post-basic child nursing science (1%), post-basic midwifery and neonatal nursing science (1%), critical care nursing: Adult (0.5%) and child psychiatric nursing science (0.5%). Only, one respondent was registered with the South African Nursing Council according to Regulation Number R1501 as Nurse Administrator (0.5%). More than two thirds of the respondents (146, 71.2%) were employed in local government facilities with these clinic staff representing a significantly higher proportion of specialist staff with 42 (58.3%) of the staff qualified in PHC nursing compared to 30 (41.7%) in the other two provincial government settings (*χ*^2^ = 8.9, *p* = 0.011) (Table 2).

**TABLE 2:** Demographics.

Variable of interest	Total respondents (*n* = 205)			Specialist in PHC (*n* = 72)			Non-specialist in PHC (*n* = 133)			Test		*p*
	*n*	%	Mean ± s.d	*n*	%	Mean ± s.d	*n*	%	Mean ± s.d	*T*	*χ* ^2^	
Age	-	-	38.5 ± 9.6	-	-	42.5 ± 8.5	-	-	36.4 ± 9.5	4.6	-	< 0.001
**Gender**	-	-	-	-	-	-	-	-	-	-	0.0	1
Male	17	8.3	-	6	8.3	-	11	8.3	-	-	-	-
Female	188	91.7	-	66	91.7	-	122	91.7	-	-	-	-
Years registered as professional nurse	-	-	11.2 ± 8.7	-	-	14.8 ± 8.3	-	-	9.3 ± 8.4	4.6	-	< 0.001
**Setting currently employed**	-	-	-	-	-	-	-	-	-	-	8.9	0.011
Provincial community health centres	43	21.0	-	22	30.6	-	21	15.8	-	-	-	-
Provincial community day centre	16	7.8	-	8	11.1	-	8	6.0	-	-	-	-
Local government clinics	146	71.2	-	42	58.3	-	104	78.2	-	-	-	-

PHC, primary health care.

### Professional nursing tasks domains: Importance, performance and training needs

Using the TNA, the standard professional nursing tasks to be performed in five domains (research and audit, communication and teamwork, clinical tasks, administration and management and supervisory tasks) were rated in terms of importance and current performance. Based on these two ratings, the training needs for each item and domain were calculated.

#### Importance of professional tasks

**FIGURE 1:**
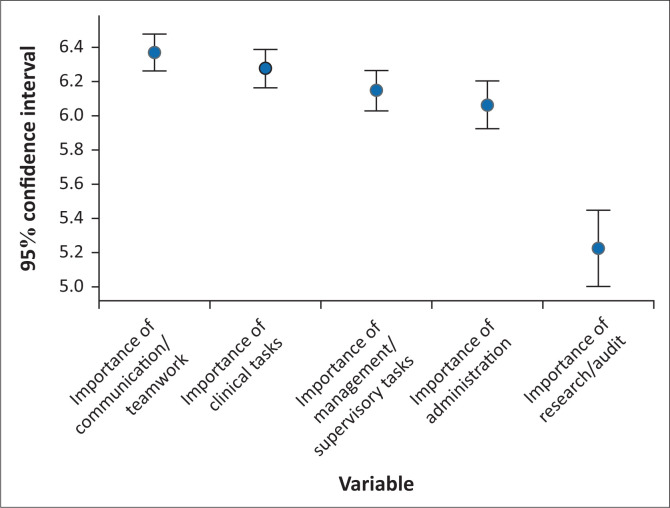
Importance ratings for professional tasks’ domains.

The respondents rated *Communication and teamwork tasks* as the most important task domain (6.38, 95% CI: 6.27–6.50]) followed by *Clinical tasks* (6.28, 95% CI: 6.16–6.40) (Figure 1). The most important individual task was a clinical task, *Treat patients* (6.56, 95% CI: 6.45–6.66), followed by two communication and teamwork tasks, *Communicating with patients face-to-face* (6.50 6.38–6.61) and *Giving information to patients* (6.48, 95% CI: 6.36–6.60) (Table 3). The research tasks as a domain were rated the least important and were rated significantly lower than the other domains (5.22, 95% CI: 5.0–5.45) (5.22, 95% CI: 5.00–5.45) (Figure 1), with the least important task identified as *Designing a research study* (4.74, 95% CI: 4.44–5.03) (Table 3).

**TABLE 3:** Tasks by importance, performance and training needs.

Subsections	Tasks	Importance		Performance		Training need	
		Mean	95%CI	Mean	95%CI	Mean	95%CI
Administration	Paperwork and/or routine data inputting	6.23	6.08–6.38	5.67	5.48–5.85	0.56	0.39–0.74
	Use technical equipment, including computers	6.02	5.84–6.21	5.12	4.89–5.36	0.90	0.66–1.15
	Undertake administrative activities	5.94	5.75–6.12	5.55	5.34–5.75	0.39	0.21–0.57
	Total	6.07	5.92–6.21	5.39	5.21–5.56	0.68	0.51–0.85
Clinical tasks	Treat patients	6.56	6.45–6.66	6.06	5.90–6.23	0.49	0.34–0.65
	Plan and organise an individual patient’s care	6.41	6.29–6.53	5.78	5.60–5.95	0.63	0.47–0.80
	Assess patients’ clinical needs	6.38	6.24–6.52	6.01	5.85–6.17	0.37	0.21–0.52
	Undertake health promotion activities	6.18	6.02–6.33	5.49	5.29–5.69	0.69	0.50–0.87
	Evaluate patients’ psychological and social needs	6.16	6.00–6.31	5.54	5.35–5.73	0.61	0.44–0.78
	Access relevant literature for your clinical work	5.98	5.80–6.16	4.87	4.62–5.13	1.08	0.83–1.34
	Total	6.28	6.16–6.40	5.57	5.42–5.72	0.70	0.56–0.85
Communication/teamwork	Communicate with patients face-to-face	6.50	6.38–6.61	6.05	5.88–6.23	0.44	0.28–0.61
	Give information to patients and/or carers	6.48	6.36–6.60	5.98	5.81–6.14	0.50	0.35–0.66
	Establish a relationship with patients	6.45	6.32–6.59	5.86	5.67–6.04	0.60	0.43–0.76
	Get on with your colleagues	6.27	6.11–6.43	5.84	5.66–6.02	0.43	0.26–0.60
	Working as a member of a team	6.26	6.10–6.43	5.93	5.75–6.12	0.33	0.16–0.50
	Provide feedback to colleagues	6.25	6.12–6.38	5.63	5.46–5.81	0.61	0.45–0.78
	Total	6.38	6.27–6.50	5.86	5.71–6.00	0.53	0.40–0.66
Management/supervisory tasks	Show colleagues and/or students how to do things	6.43	6.30–6.56	5.93	5.74–6.11	0.50	0.33–0.68
	Organise your own time effectively	6.24	6.10–6.39	5.62	5.44–5.80	0.62	0.45–0.80
	Personally cope with change in the health service	6.21	6.07–6.35	5.74	5.56–5.92	0.47	0.29–0.64
	Introduce new ideas at work	6.10	5.94–6.26	5.44	5.24–5.64	0.65	0.48–0.83
	Make do with limited resources	5.97	5.78–6.15	5.58	5.38–5.78	0.39	0.19–0.58
	Appraise your own performance	5.93	5.74–6.11	5.34	5.14–5.55	0.59	0.42–0.75
	Total	6.14	6.02–6.27	5.55	5.40–5.70	0.59	0.45–0.73
Research/audit	Apply research results to your own practice	5.56	5.32–5.80	4.48	4.20–4.76	1.08	0.82–1.34
	Interpret your own research findings	5.44	5.19–5.69	4.25	3.96–4.55	1.19	0.93–1.45
	Access research resources (e.g. time, money, etc.)	5.39	5.15–5.63	4.68	4.41–4.95	0.71	0.44–0.97
	Collect and collate relevant research information	5.25	4.98–5.52	4.13	3.84–4.43	1.12	0.84–1.40
	Statistically analyse your own research data	5.20	4.92–5.47	4.03	3.74–4.32	1.17	0.87–1.46
	Critically evaluate published research	5.19	4.93–5.45	3.98	3.70–4.26	1.23	0.95–1.51
	Identify viable research topics	5.19	4.92–5.46	4.05	3.76–4.34	1.13	0.86–1.41
	Write reports of your research studies	5.14	4.86–5.41	3.90	3.60–4.20	1.26	0.96–1.57
	Design a research study	4.74	4.44–5.03	3.66	3.36–3.96	1.07	0.76–1.38
	Total	5.22	5.00–5.45	4.13	3.89–4.37	1.21	0.96–1.47

In comparing specialist in PHC respondents with non-specialist in PHC respondents, no significant differences were reported in the importance of these domains. However, differences were recorded between local and provincial government respondents with provincial government respondents rating the importance of all domains higher and with the importance of the research domain rated significantly higher (5.67 ± 1.3 vs 5.03 ± 1.7, *U* = 2.4, *p* = 0.019).

#### Performance of professional nursing tasks

**FIGURE 2:**
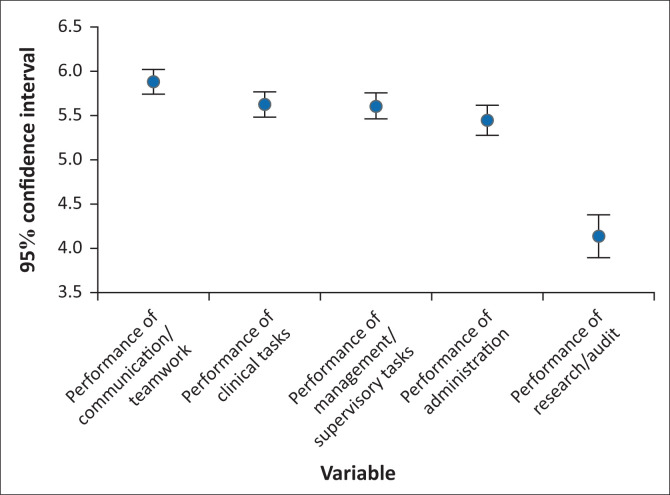
Performance ratings for professional tasks’ domains.

Performance was on average rated lower than importance. *Communication and Teamwork Tasks* had the highest performance ratings (5.86, 95 CI: 5.71–6.00), followed by *Clinical Tasks* (5.57, 95 CI: 5.42–5.72) (Figure 2). The task with the highest performance rating was the clinical task, *Treat Patients* (6.06, 95% CI: 5.90–6.23), followed by a communication and teamwork task, *Communicating with Patients face-to-face* (6.05 95% CI: 5.88–6.23), and then the clinical task *Assess Patients’ Clinical Needs* (6.01, 95% CI: 5.85–6.17) (Table 3). Performance in research tasks as a domain was again rated the lowest and was rated significantly lower than the other domains (4.13, 95% CI: 3.89–4.37]) (Figure 2), with the least important task identified as *Designing a Research Study* (3.66, 95% CI: 3.36–3.96]) (Table 3).

In comparing specialist in PHC respondents with non-specialist in PHC respondents, significant differences were reported in the performance of communication and teamwork (5.69 ± 1.1 vs 5.99 ± 1.0, *U* = 2.1, *p* = 0.032) and administration (5.20 ± 1.2 vs 5.58 ± 1.2, *U* = 2.1, *p* = 0.035). No significant differences in ratings of performance of the research, clinical tasks and management domains were recorded for specialist in PHC respondent’s versus non-specialist in PHC respondents. Differences were recorded between local and provincial government respondents, with local government respondents rating the performance of clinical tasks domains higher than provincial government respondents (5.73 ± 1.0 vs 5.38 ± 1.1, *U* = 1.9, *p* = 0.046).

#### Training needs for professional nursing tasks

**FIGURE 3:**
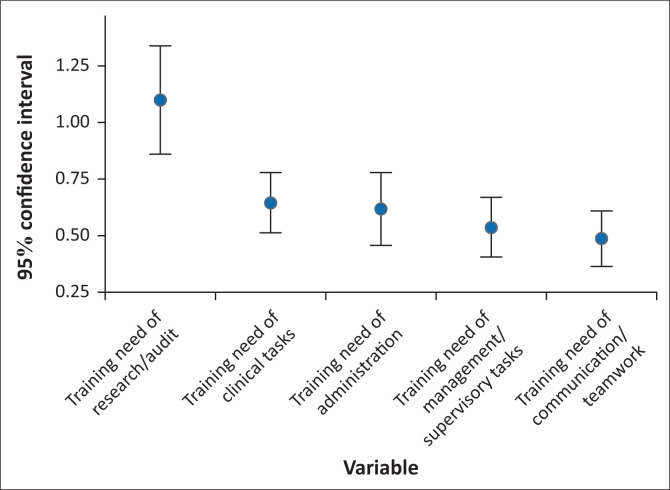
Calculated training needs for professional nursing tasks per domain.

The calculated training needs for professional nursing tasks demonstrated that the highest training need was *Research Tasks* (1.21, 95% CI: 0.96–1.47), which was significantly higher than the other calculated training needs (Figure 3), followed by *Clinical Tasks* (0.70, 95% CI: 0.56–0.85), not significantly different from the other domains. The lowest calculated training need was *Communication and Teamwork* (0.53, 95% CI: 0.40–0.66), although this was not significant (Figure 3).

Significant higher training needs were calculated for specialist in PHC respondents compared to non-specialist in PHC respondents in the research (1.46 ± 2.0 vs 0.90 ± 1.6, *U* = 1.6 *p* = 0.10), communication and teamwork (0.67 ± 0.9 vs 0.39 ± 0.8, *U* = 1.6 *p* = 0.10), clinical (0.85 ± 1.0 vs 0.53 ± 0.9, *U* = 2.1 *p* = 0.004), administration (0.82 ± 1.2 vs 0.51 ± 1.1, *U* = 1.7 *p* = 0.09) and management domains (0.70 ± 1.0 vs 0.45 ± 0.9, *U* = 1.3 *p* = 0.18). Similarly, when comparing calculated training needs, provincial government respondents had significant higher training needs in all categories compared to local government respondents, research (1.77 ± 2.1 vs 0.80 ± 1.5, *U* = 3.3 *p* < 0.001), communication and teamwork (0.70 ± 0.9 vs 0.39 ± 0.9, *U = 2.8 p* = 0.005), clinical (0.93 ± 1.0 vs 0.52 ± 0.9, *U* = 3.4 *p* < 0.001), administration (0.99 ± 1.2 vs 0.45 ± 1.1, *U* = 3.5 *p* < 0.001) and management (0.84 ± 1.1 vs 0.40 ± 0.9, *U* = 3.5 *p* < 0.001).

### Professional tasks by importance, performance and training need

In assessing the training needs associated with individual tasks, the top three tasks with the highest training needs were in the research domain with *Writing a Research Report* (1.26, 95% CI: 0.96–1.57), *Critically Evaluating Published Research* (1.23, 95% CI: 0.95–1.51) and *Interpreting Research Findings* (1.19, 95% CI: 0.93–1.45) (Table 3). Within the clinical domain, the highest rated training need was *Accessing relevant literature for Clinical Work* (1.08, 95% CI: 0.83–1.34), followed by *Undertaking Health Promotion Activities* (0.69, 95% CI: 0.50–0.87). In the management and supervisory domain, the highest rated training needs were *Introducing New Ideas at Work* (0.65, 95% CI: 0.48–0.83) (0.65, 95% CI: 0.48–0.83) and *Organising your own time effectively* (0.62, 95% CI: 0.45–0.80). In the communication and teamwork domain, *Providing Feedback to Colleagues* (0.61, 95% CI: 0.45–0.78) and *Establishing Relationships with Patients* (0.60, 95% CI: 0.43–0.76) were the highest rated training needs. Lastly, in the administrative domain, the highest rated training need was *Using Technical Equipment Including Computers* (0.90, 95% CI: 0.66–1.15).

### Specific training needs for primary health care

#### Specific contextual training domains and topics for primary health care

Based on all the training needs’ topics, irrespective of ranking, 362 topics were listed, grouped into nine training domains (Table 4). Child mental health training topics were most frequently listed (*n* = 110, 30.4%) with specific topics requested in child mental health skills and management (*n* = 48, 43.6%), child mental health (general) (*n* = 33, 30.0%), child mental health problems and disorders (*n* = 17, 15.5%) and child mental health screening and assessment (*n* = 12, 10.9%) (Table 4). This was followed by mental health (*n* = 70, 19.3%), HIV/AIDS and TB (42, 11.6%) and PHC (*n* = 41, 11.3%) topics. In the top three topics in each of the domains, skills and management was the most frequently identified (Table 4).

**TABLE 4:** Specific needs for primary health care.

Training domains	Topics (*n* = 362)	Specific topics/group	
		*n*	%
Child mental health (*n* = 110, 30.4%)	Skills and management	48	43.6
	Child mental health (general)	33	30.0
	Mental health problems and disorders	17	15.5
	Screening and assessment	12	10.9
Mental health (*n* = 70, 19.3%)	Skills and management	26	37.1
	Screening and assessment	19	27.1
	Mental health problems and disorders	13	18.6
	Mental health (general)	12	17.1
HIV/AIDS and TB (*n* = 42, 11.6%)	Skills and management	26	61.9
	HIV/AIDS/TB (general)	16	38.1
PHC (*n* = 41, 11.3%)	Screening and assessment	11	26.8
	PHC (general)	10	24.4
	Pharmacology	8	19.5
	PHC illnesses	8	19.5
	Skills and management	4	9.8
Child health (*n* = 29, 8.0%)	Child health (general)	13	44.8
	Skills and management	10	34.5
	Screening and assessment	4	13.8
	Child health legislation	2	6.9
Professional tasks (excluding clinical) (*n* = 26, 7.2%)	Nursing management	11	42.3
	Information communication and technology	8	30.8
	Nursing research	5	19.5
	Nursing education	2	7.7
Women and reproductive health (*n* = 22, 6.1%)	Skills and management	8	36.4
	Women and reproductive health (general)	8	26.4
	Screening and assessment	6	27.3
Emergencies (*n* = 15, 4.1%)	Skills and management	10	66.7
	Emergencies (general)	5	33.3
Other clinical areas (*n* = 7, 1.9%)	Intensive care	2	28.6
	Theatre	2	28.6
	Eye care	1	14.3
	Dermatology	1	14.3
	Orthopaedics	1	14.3

PHC, primary health care; HIV, human immunodeficiency virus; AIDS, acquired immunodeficiency syndrome; TB, tuberculosis.

### Top ranked specific primary health care training needs

#### Ranked primary health care training domains and topics

In ranking the specific training topics for PHC (*n* = 130), all training topics were clinical, except for professional tasks which ranked seventh. The highest ranked training domain was again for child mental health topics in the child mental health domain which was rated as most important by 47 (36.2%) respondents, followed by mental health (*n* = 20, 15.4%) and HIV/AIDS and TB (*n* = 16, 12.3%) (Table 5). The traditional PHC domains of PHC and women and reproductive health were rated higher by the non-specialist in PHC respondents than the specialist in PHC respondents (*n* = 5, 6.1% vs *n* = 1, 2.1%). However, both groups rated child mental health and mental health as the most important (Table 5).

**TABLE 5:** Top ranked specific primary health care training needs.

Domains	All (*n* = 130)		Specialist in PHC (*n* = 48, 37%)		Non-specialist in PHC (*n* = 82, 63%)	
	*n*	%	*n*	%	*n*	%
1. Child mental health	47	36.2	18	37.5	29	35.3
2. Mental health	20	15.4	7	14.6	13	15.9
3. HIV/AIDS and TB	16	12.3	5	10.4	11	13.4
4. Child health	13	10.0	5	10.4	8	9.8
5. PHC	10	7.7	3	6.3	7	8.5
6. Emergency	9	6.9	2	4.2	7	8.5
7. Professional tasks (excluding clinical)	7	5.4	5	10.4	2	2.4
8. Women and reproductive health	6	4.6	1	2.1	5	6.1
9. Other clinical areas	2	1.5	2	4.2	0	0.0

PHC, primary health care; HIV, human immunodeficiency virus; AIDS, acquired immunodeficiency syndrome; TB, tuberculosis.

## Discussion

This is the first study to investigate the training needs of professional nurses working in PHC facilities in the Cape Metropole, Western Cape, South Africa. The first finding of note was the low level of specialisation (35.1%) in PHC Nursing, which is an additional qualification in Clinical Nursing Science, Health Assessment, Treatment and Care, in South Africa. Although professional nurses render most of the healthcare at PHC level, professional nurses qualified in Clinical Nursing Science, Health Assessment, Treatment and Care are in short supply.^33^ This study supports the reported current shortage of professional nurses who qualified for the specialist qualification in Clinical Nursing Science, Health Assessment, Treatment and Care, with only 5436 who registered with the South African Nursing Council over the last five years (2017–2021).^34^ This negatively influences the provision of effective patient care and the achievements of local or national population health goals such as the new proposed National Health Insurance.^33^

The second finding based on the TNA was that the respondents rated the *Communication and Teamwork* domain as the most important and highest performance domain and the research domain significantly lower than the other domains, with the least important task identified as designing a research study. Although this study had 25.8% of respondents with a degree and 42% of respondents with a post-basic qualification in any clinical nursing specialty such as post-basic psychiatric nursing science, Occupational health nursing, post-basic child nursing science, post-basic midwifery and neonatal nursing science, Critical Care Nursing: Adult and Child psychiatric nursing science, the self-rated low importance and performance of designing research studies by the respondents is of concern. This is a concern as research methodologies are offered as an introduction to research during an undergraduate nursing degree as well as at post-basic diploma levels in South Africa. Furthermore, according to the South African Nursing Council Competency Framework for PHC nurse specialists, performance of research is a core competency.^35^ Our findings in this study are in keeping with an earlier study^26^ which found that nurses rated importance and performance in designing research studies lower in comparison with other healthcare professionals, such as allied health professionals and doctors. A reasonable explanation for low performance of research by PHC nurse specialists in this study may be due to an increased workload^9^ that may negatively impact time management.^36^

The low rating of importance and performance in the research task domain in this study, resulted in research being identified as the highest training need by PHC nurses. The results of this study are comparable with the results from an earlier study^22^ among professional nurses employed in designated PHC facilities in Greece and confirmed that most of the training needs of PHC nurses’ tasks stem from research and or audit tasks.^22^ Our results appear to be well substantiated by other researchers who assert that poor recognition of importance and performance of research among professional nurses employed in designated PHC facilities could possibly be due to their non-involvement in research as undergraduate nursing students.^24^ Nonetheless, the inability of professional nurses employed in the designated PHC facilities to recognise the importance of research by nurses is not a new debate. For instance, in a study conducted among family planning nurses in England and Wales, the research section was completely omitted as being part of their occupational profile as a nurse prescriber.^24^ An additional note was that in our study, significantly higher training needs were calculated for specialists in PHC respondents compared to non-specialist in PHC respondents in the research, communication and teamwork, clinical and administration. A possible explanation for this finding is because specialist PHC respondents may be more aware of these domains as they are part of the core content in the post-basic specialisation in PHC.^37^ Likewise, when comparing calculated training needs, provincial government respondents had significantly higher training needs in all categories compared to local government respondents. A possible explanation for this finding could be due to provincial government respondents focus on a variety of complex health problems across the age spectrum^29^ and may, therefore, request training in all categories to stay up to date with the latest developments in healthcare.

In determining the specific training topics in PHC, most were clinical training topics specifically related to current practice in PHC, with professional topics only ranking seventh in importance. In both the most frequently listed topics and the most important ranking of training domains, child mental health was listed as the most important training need facing PHC nurses in practice. Specific topics listed were child mental health nursing skills and management, child mental health problems and disorders and child mental health screening and assessment. These findings are well supported by a recent study^8^ that focused on a situational analysis of child and adolescent mental health services and systems in the Western Cape Province of South Africa, which found that there were no available data on child mental health training for professional nurses, despite these researchers acknowledging that they provided training sessions such as workshops and seminars to all PHC staff.^8^ Another study conducted among professional nurses in PHC clinics in KwaZulu-Natal, South Africa, also reported a lack of knowledge in the management of patients with mental health problems.^38^ Furthermore, in South Africa, the PHC and the Child Nursing curriculum reflected that the content of PHC and the Child Health Nursing Programmes in South Africa mostly focus on common physical illnesses such as HIV/AIDS,^39^ childhood malnutrition, respiratory tract infections and diarrhoea.^40^ This highlights a major gap in both the PHC and the Child Health Nursing curriculum in South Africa and provides a focus for continuous professional development in the near future.

### Strengths and limitations

The findings of the study provide baseline information on the training needs of PHC nurses in the Cape Metropole, Western Cape, South Africa, a topic that has been neglected to date. Furthermore, the study is timely as it will assist with the development of short courses for CPD and contribute to the current process of review and alignment of nursing curricular to the Higher Education Qualification Sub-Framework (HEQSF) in South Africa. The study has some limitations. Firstly, because the Hennessy–Hicks TNA questionnaire is largely a self-reported questionnaire, professional nurses in PHC rated the importance of all the tasks and their own performance of these tasks which may be subjective and not a true reflection of the PHC nurses’ training needs. Secondly, the study was conducted among professional nurses employed in two of the four health substructures in the Western Cape. Therefore, the results of this study cannot be generalised to the broader nursing fraternity. Lastly, a better response rate could have been obtained; however, due to COVID-19 pandemic, some professional nurses were on sick leave or in isolation during the data collection period.

### Recommendations

Firstly, the engagement of PHC nurses in collaborative research has the potential to improve nursing care and patient outcomes through evidence-based practice.^41^ Primary health care nurses must be encouraged to initiate and carry out research in clinical areas, and other strategies such as incentives for nurses and publicly acknowledging PHC nurses who conduct research in the clinical service areas can be used as motivation.^41^ Secondly, the learning outcomes in both undergraduate and postgraduate nursing programmes that focus on research should receive as much attention as all other learning outcomes in these programmes. In the absence of a formal CPD programme for nurses and midwives in South Africa, and the omission of child mental health in the recent CPD Framework,^20^ nursing education institutions must (1) develop short courses and integrate child mental health nursing in their undergraduate nursing programmes to prepare non-specialist PHC nurses to function more comprehensively and (2) develop specialist child mental health nursing postgraduate diploma programmes to ensure that future nurses working in PHC are competent and have adequate knowledge in the screening and assessment of children for mental health problems., Thirdly, nurse managers employed in PHC must identify the training needs of staff during their first performance management meeting and follow up every 3 months on progress towards accomplishing the identified training needs. Lastly, the study should be replicated in future and should expand beyond the two health substructures in the Western Cape and include rural and different health settings (i.e. secondary and tertiary hospitals) in South Africa. When replicating the study, all ratings (A, B, C and D) of the Hennessy–Hicks TNA questionnaire should be included. Furthermore, a qualitative study should be conducted to gain a deep understanding of the training needs of PHC nurses.

## Conclusion

The aim of this study was to determine the training needs of professional nurses working in PHC facilities in the Cape Metropole, Western Cape, South Africa. The survey highlights the low level (35.1%) of specialisation as PHC and identified research and child mental health as the most important professional and clinical training needs.
